# [^18^F]ML-10 PET: Initial Experience in Glioblastoma Multiforme Therapy Response Assessment

**DOI:** 10.18383/j.tom.2016.00175

**Published:** 2016-12

**Authors:** Matthew J. Oborski, Charles M. Laymon, Frank S. Lieberman, Yongxian Qian, Jan Drappatz, James M. Mountz

**Affiliations:** 1 Department of Bioengineering, University of Pittsburgh, Pittsburgh, Pennsylvania;; 2 PET Center, Department of Radiology, University of Pittsburgh, Pittsburgh, Pennsylvania;; 3 Department of Neurology and Medicine, University of Pittsburgh, Pittsburgh, Pennsylvania; and; 4 MR Research Center, Department of Radiology, University of Pittsburgh, Pittsburgh, Pennsylvania

**Keywords:** [^18^F]ML-10, imaging biomarker, GBM, early-therapy assessment

## Abstract

The ability to assess tumor apoptotic response to therapy could provide a direct and prompt measure of therapeutic efficacy. ^18^F-labeled 2-(5-fluoro-pentyl)-2-methyl-malonic acid ([^18^F]ML-10) is proposed as a positron emission tomography (PET) apoptosis imaging radiotracer. This manuscript presents initial experience using [^18^F]ML-10 PET to predict therapeutic response in 4 patients with human glioblastoma multiforme. Each patient underwent [^18^F]ML-10 PET and contrast-enhanced magnetic resonance imaging (MRI) before (baseline) and at ∼2–3 weeks after therapy (early-therapy assessment). All PET and MRI data were acquired using a Siemens BioGraph mMR integrated PET/MRI scanner. PET acquisitions commenced 120 minutes after injection with 10 mCi of [^18^F]ML-10. Changes in [^18^F]ML-10 standard uptake values were assessed in conjunction with MRI changes. Time-to-progression was used as the outcome measure. One patient, ML-10 #4, underwent additional sodium-23 (^23^Na) MRI at baseline and early-therapy assessment. Siemens 3 T Magnetom Tim Trio scanner with a dual-tuned (^1^H-^23^Na) head coil was used for ^23^Na-MRI, acquiring two three-dimensional single-quantum sodium images at two echo times (TE). Volume-fraction-weighted bound sodium concentration was quantified through pixel-by-pixel subtraction of the two single-quantum sodium images. In the cases presented, [^18^F]ML-10 uptake changes were not clearly related to time-to-progression. We suggest that this may be because the tumors are undergoing varying rates of cell death and growth. Acquisition of complementary measures of tumor cell proliferation or viability may aid in the interpretation of PET apoptosis imaging.

## Introduction

An array of radiotracers is available that allow the characterization of various tumor tissue aspects including apoptosis (1, 2). Apoptosis imaging is particularly attractive in the context of therapy response assessment because it may provide a direct and prompt measure of therapy effect. In addition to providing an indicator of effective therapy, apoptosis imaging may prove useful for identifying a time-point at which a therapy is no longer effective, thus providing the opportunity for timely management changes. Nevertheless, interpretation of apoptosis imaging data is complicated by the transient nature of the process. Furthermore, although a measure of therapy-induced apoptosis provides important information about the therapy effect, it does not provide a complete picture of the tumor status, which depends on both proliferation and apoptosis rates.

^18^F-labeled 2-(5-fluoro-pentyl)-2-methyl-malonic acid ([^18^F]ML-10) provides one approach for imaging therapy-induced apoptosis by identifying cells that have permanently depolarized cell membranes, a signature of apoptosis (3, 4). *In vitro* studies in which Jurkat cells (human adult leukemia T-lymphocyte cells) were incubated with anti-Fas antibody showed a correlation between [^3^H]ML-10 accumulation and known apoptotic markers such as caspase-3 activity, mitochondrial membrane depolarization, and phosphatidylserine externalization (3, 5). Moreover, simultaneous incubation of Jurkat cells with anti-Fas antibody and Z-VAD-FMK (Z-Val-Ala-Asp-fluoromethyl ketone), a pan-caspase inhibitor, showed similar levels of [^3^H]ML-10 accumulation to untreated control cells, demonstrating that significant [^3^H]ML-10 accumulation can be prevented by inhibiting caspase activation (3).

In vivo, preclinical studies in a mouse stroke model showed selective radioactivity accumulation at the site of the infarct region on positron emission tomography (PET) at 60 minutes after injection with [^18^F]ML-10 (6). Correlative [^18^F]ML-10 Phosphorimaging of ex vivo brain sections confirmed the accumulated [^18^F]ML-10 to be localized in the infarction region (6). Furthermore, analysis of tissue samples taken from excised brain sections confirmed via terminal deoxynucleotidyl transferase nick end labeling (TUNEL) staining that cells in the region of [^18^F]ML-10 accumulation were undergoing apoptosis (6).

In healthy humans, [^18^F]ML-10 exhibits rapid clearance from blood and normal tissue, with low tracer metabolism and no defluorination (7). In particular, an analysis of plasma samples from 8 human subjects found a 97.5% ± 0.4% unchanged [^18^F]ML-10 fraction 150 minutes after injection (7). In the same study, selective accumulation of [^18^F]ML-10 was observed on PET in the testes of male humans and mice (7), a known site of apoptosis as a result of processes related to spermatogenesis (8). Further investigation of this phenomenon using fluorescent microscopy imaging revealed that cells exhibiting dansyl-ML-10 fluorescence in the testicular tissue of male mice were positive for characteristic apoptotic DNA fragmentation assessed via TUNEL staining (7).

In a prior published report from our group, Oborski et al. (9) performed TUNEL staining on tumor tissue from a human glioblastoma multiforme (GBM) subject obtained via fine-needle magnetic resonance imaging (MRI) guided stereotactic biopsy. The results revealed the presence of cells undergoing apoptosis, which correlated with baseline [^18^F]ML-10 uptake at the tumor site.

In this report, we show the possible utility and difficulties in apoptosis imaging in 4 example patients with GBM using [^18^F]ML-10. The patients were imaged at 2 imaging time-points: baseline (BL; before therapy initiation) and early-therapy assessment (ETA; ∼2–3 weeks after therapy initiation). In one case, we present correlative scans from sodium-23 (^23^Na) magnetic resonance (MR) imaging. Changes in intracellular sodium concentration are hypothesized to be indicative of changes in tumor cell density (10).

## Methods and Materials

### Subjects

Four patients with newly diagnosed or recurrent, histologically confirmed GBM were enrolled in this study. Table 1 contains patient demographics, therapy received, and the time-to-progression (TTP; assessed on patients' contrast-enhanced MRI [CE-MRI]) in months. The imaging study was designed such that each patient underwent a total of two scanning sessions: BL (prior to therapy initiation) and ETA (∼2–3 weeks after therapy initiation). PET and MRI data were acquired at each time-point. At the time of enrollment, therapy for patients ML-10 #3 and ML-10 #4 included fractionated external beam radiation therapy (RT) to a total dose of 60 Gy, in daily 2 Gy fractions, with concomitant temozolomide (TMZ) at a dose of 75 mg/kg/day, followed by adjuvant TMZ at 150–200 mg/m^2^/d on days 1–5 of 28-day cycles for 12 months. Patient ML-10 #1 received the same TMZ regimen as patients ML-10 #3 and ML-10 #4, but did not receive RT. ML-10 #2 (recurrent GBM) was treated initially on Radiation Therapy Oncology Group Protocol 0825, receiving external beam fractionated radiation over 42 days with concurrent TMZ followed by 12-monthly cycles of TMZ. Bevacizumab (10 mg/kg) was injected in the patient's arm every 14 days beginning on day 21 of RT. The tumor progressed after the 10th cycle of TMZ, and he entered a clinical trial of ANG1005, a taxane derivative. This subject's BL imaging study was performed before the first cycle of ANG1005, but after 1 cycle, there was further symptomatic progression, and bevacizumab administration was restarted at a dose of 10 mg/kg.

**Table 1.** Subject Demographics and Treatment

Subject ID	Age (Years)	Gender (M/F)	GBM Type	Therapy	BL PET (Days Before Therapy Initiation)	ETA PET (Days After Therapy Initiation)	TTP (Months)
ML-10 #1	72	M	Newly diagnosed	TMZ	0	11	2
ML-10 #2	48	M	Recurrent	ANG1005	10	24	<1
ML-10 #3	60	M	Newly diagnosed	RT + TMZ	5	16	25
ML-10 #4	56	M	Newly diagnosed	RT + TMZ	3	15	18

This study was approved by the Institutional Review Board of the University of Pittsburgh, and written consent forms were signed by the patients.

### Radiochemical Preparation of [^18^F]ML-10

[^18^F]ML-10 was produced at the University of Pittsburgh PET Facility Radiochemistry Laboratory (Pittsburgh, Pennsylvania) in accordance with methods and procedures contained within U.S. Food and Drug Administration Investigational New Drug Application #106662 (*Sponsor Investigator:* James M. Mountz, MD, PhD). In brief, [^18^F]fluoride delivered from the cyclotron was trapped in a quaternary ammonium solid-phase extraction cartridge (Accell Plus QMA (Waters Corporation, Milford, MA, USA); initially treated with 10 mL 0.1M aqueous sodium bicarbonate followed by 5-mL anhydrous acetonitrile followed by air). An aqueous solution of 22 mg K_2_CO_3_ and 7 mg Kryptofix222 (Sigma-Aldrich, Milwaukee, WI, USA) (1.0 mL total volume) was passed through the cartridge to elute the [^18^F]fluoride. Azeotropic drying at 90°C in a reaction vial under argon with acetonitrile (2 × 1 mL) furnished the dry [^18^F]fluoride complex to be used for the fluorination reaction (11).

A solution of ML-10 precursor (2–3 mg) in anhydrous acetronitrile (1 mL) was heated at 100°C for 15 minutes. The crude reaction mixture was cooled and aqueous hydrochloric acid (2 N, 0.5 mL) was added. The solution was allowed to react at 110°C for 15 minutes. [^18^F]ML-10 isolation was achieved via purification using a semipreparative reversed-phase high-performance liquid chromatography (HPLC) system (mobile phase: water/acetonitrile/trifluoroacetic acid 80/20/1 [v/v/v], Phenomenex ODS [octadecyl; C-18] column (Phenomenex, Torrance, CA, USA) flow rate: 4 mL/min; and time 0–5 minutes; flow was then increased to 7 mL/min for the remainder of the purification process) to provide purified [^18^F]ML-10. The HPLC product fraction containing [^18^F]ML-10 (RT ∼25 minutes) was diluted with 0.1 N hydrochloric acid (60 mL) and passed across a C-18 Sep Pak cartridge (Waters Corporation, Milford, MA, USA). The final product was recovered into a sample vial by slowly flushing the C-18 Sep-Pak cartridge with absolute ethanol (1 mL).

Chemical purity, radiochemical purity, and specific activity were assessed by analytical HPLC (Phenomenex Prodigy ODS-3 5 micron [4.6 × 250 mm]; mobile phase: 25/75/0.1 MeCN/H_2_O/trifluoroacetic acid (v/v/v); flow rate: 1.5 mL/min; and λ = 205 nm). To prepare a solution suitable for in vivo administration, the 1-mL ethanol preparation containing [^18^F]ML-10 was passed through a sterilization filter (Millipore FG (Millipore, Cork, Ireland), 0.22 μm, 25 mm) into a 20-mL sterile empty vial. The ethanol solution was diluted with saline for injection; United States Pharmacopeia (USP) to provide a final product formulation suitable for intravenous administration. [^18^F]ML-10 was obtained in overall yields of 35% ± 10% (decay-corrected). Radiochemical and chemical purities were >95%, and the end-of-synthesis specific activity was >37 GBq/μmol (1000 Ci/mmol).

### PET/MR Imaging

PET and structural MR imaging were performed simultaneously on a Siemens integrated PET/MR scanner (Biograph mMR, Siemens Medical Solutions, Erlangen, Germany). PET acquisitions were started 120 minutes after injection of 10 mCi of [^18^F]ML-10 and continued for 30 minutes at 5 min/frame. Each data frame was reconstructed (using filtered backprojection) into an image with a matrix size 128 × 128 × 127 (axial) and a voxel size of 2.24 × 2.24 × 2.03 mm^3^ using manufacturer's software. Corrections for attenuation, radioactive decay, scatter, random coincidences, and dead time were included. The frame images were examined for motion, and, when required, the frames were motion-corrected on a frame-by-frames basis. Structural imaging included CE T1-weighted MRI.

### Sodium-23 MRI

One patient (ML-10 #4) underwent sodium-23 (^23^Na) MRI that was performed on a 3T MRI scanner with multinuclear capability (Magnetom Tim Trio, Siemens Medical Solutions, Erlangen, Germany) using a dual-tuned (^1^H-^23^Na) head coil (Advanced Imaging Research, Cleveland, Ohio). Sodium data acquisition started ∼15 minutes after completion of the [^18^F]ML-10 PET scan, and continued for 22 minutes during which two three-dimensional single-quantum (SQ) sodium images were acquired, one with an ultrashort TE of 0.5 milliseconds and the other with a short TE of 5.0 milliseconds. Sodium data acquisition used a custom-developed pulse sequence (twisted projection imaging) (12) with an enhanced signal-to-noise ratio. The following were the acquisition parameters: field of view = 220 mm, matrix size = 64 × 64 × 64, 3-dimensional isotropic nominal resolution = 3.44 mm, flip angle = 80° (limited by specific absorption rate), relaxation time = 100 milliseconds, and averages = 4. Data were corrected for *B*_0_-field inhomogeneity and T2* decay using custom-developed software.

The two SQ-images were subtracted in magnitude to generate an image of bound sodium (13). Quantification of total sodium concentration (TSC) and volume-fraction-weighted bound sodium concentration (vBSC) was implemented pixel-by-pixel on the SQ and subtraction images through an integrated calibration, such that the resulting TSC and vBSC images were in millimolar units. Technical details of the bound sodium MRI and associated quantification of sodium concentrations are reported in Qian et al.'s study (13).

### Image Processing

For each [^18^F]ML-10 PET acquisition, the 5 motion-corrected time frames were averaged to produce a single framed, “static,” image. Static images were then converted into units of standard uptake values (SUV) over the entire field of view using the commercially available software PMOD 3.6 (University Hospital Zurich, Zurich, Switzerland). Each voxel SUV was computed using the following equation:

$$\text{SUV}=\frac{\text{Voxel Activity}}{\left(\frac{\text{Injected Dose}}{\text{Subject Weight}}\right)},$$

where the injected dose and voxel activity are each decay-corrected to the start time of the PET scan. In the following, SUV_max_ denotes the maximum SUV in the tumor region.

All figures presented in the results were generated using MIM 5.4 image analysis software (MIM Software Inc., Cleveland, Ohio).

## Results

### Case Example: Increased [^18^F]ML-10 SUV Between BL and ETA With an Increase in Tumor Size on CE-MRI

**Figure 1.**
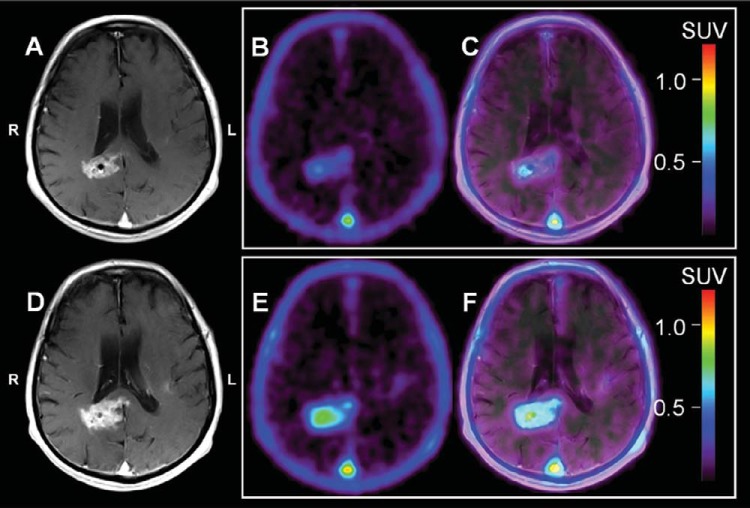
Patient ML-10 #1. Representative baseline (BL; A–C) and early-therapy assessment (ETA; D–F) contrast-enhanced (CE) magnetic resonance imaging (MRI), [^18^F]ML-10 standard uptake value (SUV), and positron emission tomography (PET)/CE-MRI fusion images for a 72-year-old male with a new glioblastoma multiforme (GBM) diagnosis. All images are shown coregistered to the patient's BL CE-MRI. BL CE-MRI (A) shows enhancement in the region of the right periventricular white matter/splenium of corpus callosum and left insular region. ETA CE-MRI (D) shows an increase in size and contrast enhancement consistent with progression. On PET, BL [^18^F]ML-10 SUV image (B) shows mild therapy-naive uptake (BL SUV_max_ = 0.54) located at the tumor site on PET/CE-MRI (C). The ETA [^18^F]ML-10 SUV image (E) shows increased [^18^F]ML-10 uptake (ETA SUV_max_ = 0.84) at the GBM site (F) consistent with increased apoptosis compared with BL.

Figure 1 shows representative BL (top row: Figure 1A–C) and ETA (bottom row: Figure 1D–F) CE-MRI and [^18^F]ML-10 PET sections for patient ML-10 #1. On PET, it is visually observed that the patient's GBM increases in SUV overall between BL and ETA (BL SUV_max_ = 0.54; ETA SUV_max_ = 0.84). On CE-MRI, the patient's GBM appears to increase in size and degree of contrast enhancement, suggesting tumor progression. Clinically, patient ML-10 #1 was interpreted to show progression at 2 months after therapy initiation (Table 1), during which time, the patient was still receiving adjuvant TMZ. Taken together, one possible explanation for these results is that although underlying cell death is occurring, proliferation is outpacing cell death, leading to a short TTP. Interpreted in this manner, this case suggests that in tumors showing a rapid increase in size on MRI, a high apoptosis signal at 2 weeks after therapy initiation may reflect regional heterogeneity with areas of increased apoptotic death in a larger rapidly proliferating tumor.

### Case Example: Decreased [^18^F]ML-10 SUV Between BL and ETA With an Increase in Contrast Enhancement on MRI

**Figure 2.**
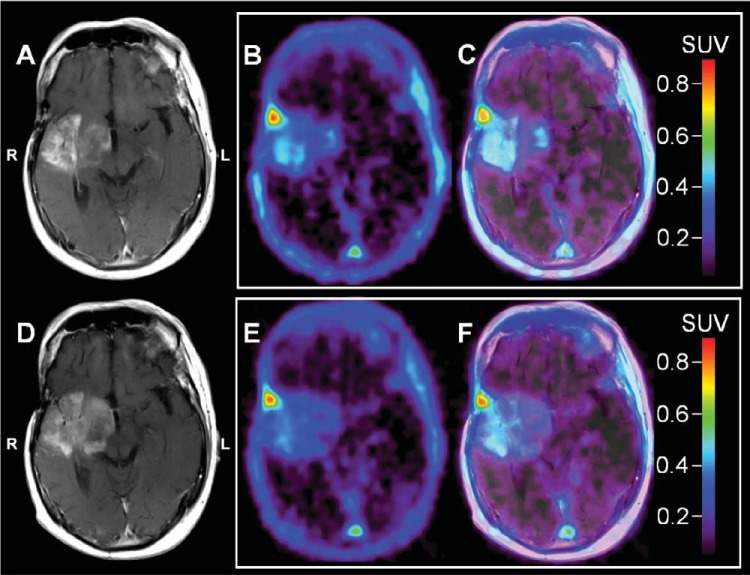
Patient ML-10 #2. Representative baseline (BL; A–C) and early-therapy assessment (ETA; D–F) contrast enhanced (CE) MRI, [^18^F]ML-10 standard uptake value (SUV), and PET/CE-MRI fusion images for a 48 years old male with a recurrent GBM diagnosis. All images are shown coregistered to the patient's BL CE-MRI. BL CE-MRI (A) shows an infiltrative mass involving portions of the right frontal and temporal lobes, with the greatest involvement in the right insular cortex anterior temporal lobe and right basal ganglia. ETA CE-MRI (D) shows progression of disease compared with BL as evidenced by increased infiltration, in particular, of the right insular region into the right basal ganglia with some increased mass effect on the third ventricle, which is displaced slightly toward the right. There is also an increase in the extent of associated enhancement and extension into the right cerebral peduncle. On PET, BL [^18^F]ML-10 SUV image (B) shows mild therapy-naive uptake (BL SUVmax = 0.54) located at the tumor site on PET/CE-MRI fusion (C). The ETA [^18^F]ML-10 SUV image (E) shows little change in [^18^F]ML-10 uptake (ETA SUV_max_ = 0.48) at the tumor site (F) consistent with minimal changes in GBM apoptosis rate compared with BL.

Imaging results for patient ML-10 #2 are illustrated in Figure 2. Visually, the patient's ETA [^18^F]ML-10 PET showed a mild overall decrease in SUV relative to BL (BL SUV_max_ = 0.54, ETA SUV_max_ = 0.48), indicating low apoptosis during the time the tumor was rapidly progressing. As seen in Table 1, this patient had a very poor outcome. Similar to the first case, these results suggest that proliferation is outpacing cell death.

### Case Example: Decreased [^18^F]ML-10 SUV Between BL and ETA With a Corresponding Decrease in Tumor Size on CE-MRI

**Figure 3.**
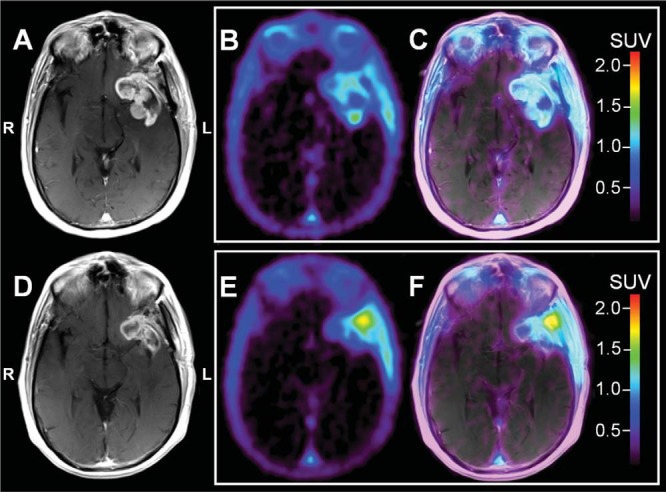
Patient ML-10 #3. Representative baseline (BL; A–C) and early-therapy assessment (ETA; D–F) contrast enhanced (CE) MRI, [^18^F]ML-10 standard uptake value (SUV), and PET/CE-MRI fusion images for a 60 year old male with a newly diagnosed left temporal GBM. All images are shown coregistered to the patient's BL CE-MRI. Before BL imaging, the patient underwent debulking. BL CE-MRI (A) shows a large residual component of peripherally enhancing, centrally cystic mass along the margins of the resection cavity within the right anterior temporal lobe, particularly involving the amygdala, and also extending anteriorly to the right orbitofrontal region. Compared with BL, the ETA CE-MRI (D) shows a reduction in CE. There is interval resolution of previously identified midline shift and sulcal effacement. On PET, the BL [^18^F]ML-10 SUV image (B) shows uptake in the CE region (C). The ETA [^18^F]ML-10 SUV image (E) shows decreased CE-associated uptake (F); however, there is increased [^18^F]ML-10 uptake in the region of the lateral border (E and F), possibly related to radiation therapy/chemotherapy associated changes.

Figure 3 shows sections for patient ML-10 #3. Before BL imaging, this patient underwent surgical debulking. Compared with BL, the ETA [^18^F]ML-10 PET showed pronounced downward change in SUV (BL SUV_max_ = 1.51; ETA SUV_max_ = 1.08) and a reduction in the extent of the [^18^F]ML-10 distribution. The reduced volume of [^18^F]ML-10 uptake is consistent with the decrease in tumor size observed on the patient's ETA CE-MRI compared with BL. A possible interpretation is that the initial reduction in [^18^F]ML-10 SUV is due to very early cell death, i.e., cell death prior to the ETA time point (TTP = 25 months).

### Case Example: PET Apoptosis Imaging Combined With Measurement of vBSC

**Figure 4.**
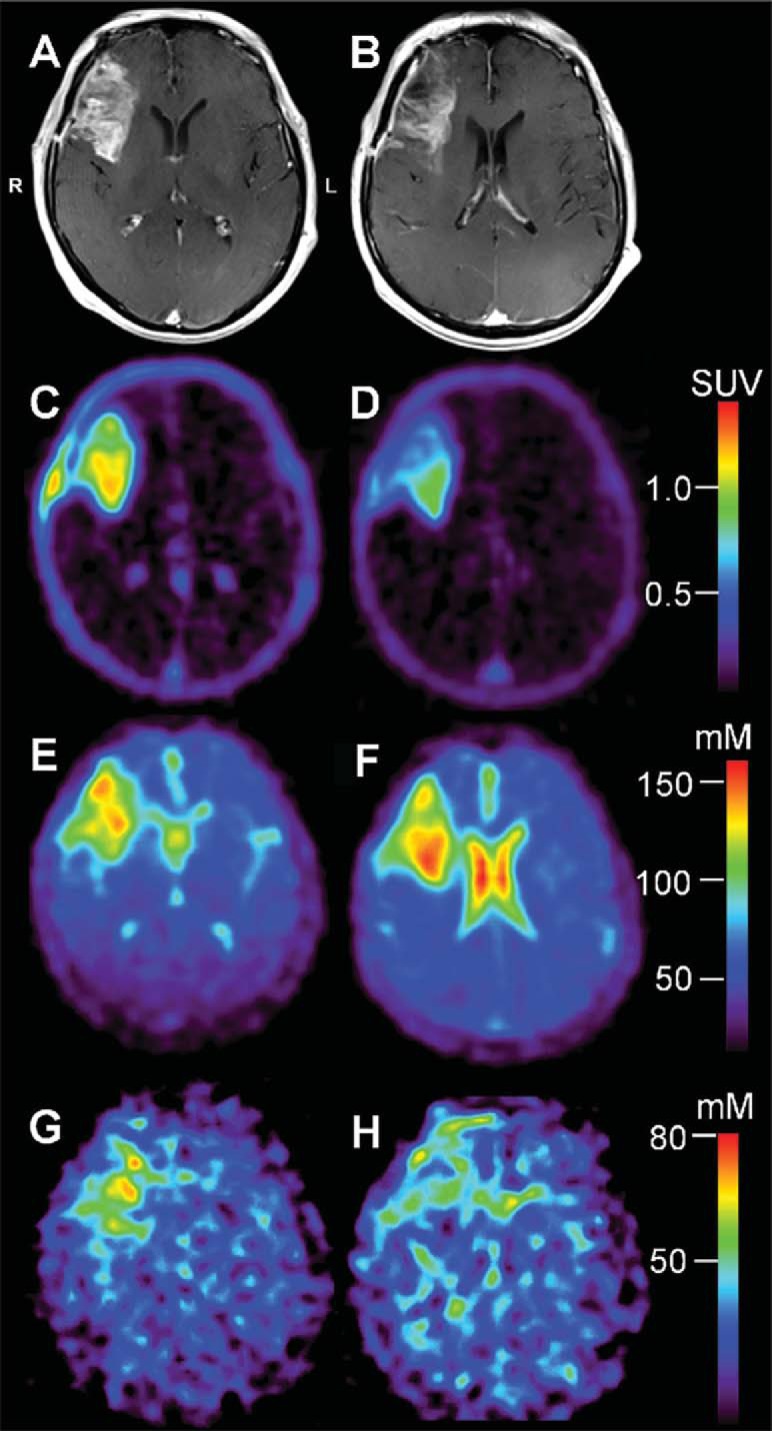
Patient ML-10 #4. Representative baseline (BL; before therapy initiation) and early-therapy assessment (ETA; 15 days after therapy initiation) contrast enhanced MRI (CE-MRI; top row, A and B, respectively), BL and ETA [^18^F]ML-10 PET (second row, C and D, respectively), BL and ETA total sodium concentration (TSC) MRI (third row, E and F, respectively), and volume-fraction-weighted bound sodium concentration MRI (vBSC-MRI; bott'm row, G and H, respectively) sections of a 56-year-old male with a new GBM diagnosis. All images are shown coregistered to the patient's BL CE-MRI. Due to a technical challenge, this subject's ETA PET was acquired 55 min later than scheduled, meaning that the imaging time frame reflects 175–205 min post injection, instead of 120–150 min post-injection, which was the protocol. At BL, the patient's CE-MRI scan (A) shows a large, peripherally enhancing and centrally necrotic intra-axial mass centered in the right frontal lobe extending to the floor of the anterior cranial fossa and involving the medial–anterior aspect of the right insula, and the right aspect of the genu of corpus. The ETA CE-MRI (B) shows a decrease in the size and contrast enhancement with anterior and central necrosis. Overall, this represents an excellent response to therapy. On PET, BL [^18^F]ML-10 SUV image (C) shows intense therapy-naive uptake (SUV_max_ = 1.41), particularly in the posterior portion of the mass. Compared with BL, the ETA [^18^F]ML-10 SUV image (D) shows a decrease in the uptake (SUV_max_ = 1.07) consistent with very low apoptosis in the anterior portion of the mass, and significant reduction in the posterior portion of the mass. At BL, the patient's TSC-MRI (E) shows high ^23^Na concentration in the CSF portion of the tumor, positioned anteriorly, with moderately increased (compared with contralesional hemisphere) TSC, posteriorly, in the viable tumor tissue. Similarly, the BL vBSC image (G) shows increased signal (compared with contralesional hemisphere) in the viable tumor tissue component. At ETA, the TSC-MRI (F) shows an increase in TSC at the region of the viable tumor tissue, with a corresponding decrease in vBSC (H) in the same location, suggesting that the increased TSC is a result of increased free sodium due to response to therapy, consistent with the patient's long time-to-progression (18 months).

Figure 4 shows representative CE-MRI (top row) and [^18^F]ML-10 SUV (second-row) sections for patient ML-10 #4. Compared with BL (Figure 4A), the ETA CE-MRI of ML-10 #4 (Figure 4B) shows a decrease in size and degree of contrast enhancement of the tumor, consistent with the patient's long TTP (18 months; Table 1). On PET, the patient's BL [^18^F]ML-10 SUV image (Figure 4C) shows initial therapy-naive accumulation, followed by a marked decrease in SUV (BL SUV_max_ = 1.41; ETA SUV_max_ = 1.07) at ETA (Figure 4D). Taken together, the patient's BL and ETA CE-MRI and PET images are interpreted as showing an initial overall reduction in [^18^F]ML-10 SUV, suggesting effective and very early cell death (within days), such that by 2 weeks, there was very little growth or death. With this interpretation, a concurrent decrease in [^18^F]ML-10 SUV and tumor size and contrast enhancement at 2 weeks, relative to pre-therapy scans, would suggest an overall long-term favorable response and long TTP. Due to a technical challenge, this subject's ETA PET was acquired 55 min later than scheduled, meaning that the imaging time frame reflects 175–205 min post injection, instead of 120–150 min post-injection, which was the protocol.

Additional data were acquired in the case of patient ML-10 #4, namely, a newly developed MR protocol aimed at measuring vBSC (14). We are investigating this quantity as a potential indicator of malignant cell concentration and anticipate that it may be a more sensitive measure of tumor viability than TSC-MRI, which is highly dependent on extracellular sodium concentration.

Representative TSC-MRI and vBSC-MRI sections are shown in Figure 4 (third and fourth rows, respectively) coregistered to the patient's BL CE-MRI. At BL, the patient's TSC-MRI (Figure 4E) shows high ^23^Na concentration in the cerebrospinal fluid (CSF) portion of the tumor, positioned anteriorly, with moderately increased (compared with contralesional hemisphere) TSC, posteriorly, in the viable tumor tissue. Similarly, the BL vBSC-MRI (Figure 4G) shows increased signal (compared with the contralesional hemisphere) in the viable tumor tissue component. At ETA, the TSC-MRI shows an increase in TSC at the region of viable tumor tissue (Figure 4F), with a corresponding decrease in vBSC (Figure 4H) in the same location, suggesting that the increased TSC is a result of increased free sodium due to response to therapy, consistent with the patient's long TTP (18 months; Table 1). Moreover, the observed decrease in vBSC coupled with an increase in TSC between imaging time-points is consistent with the hypothesis that the reduction in [^18^F]ML-10 SUV between BL and ETA is a result of reduced tumor cell density due to effective therapy, and, therefore, an overall reduction in tumor apoptosis.

## Discussion

One advantage that [^18^F]ML-10 may have in terms of response assessment in GBM is that ML-10 accumulation is specific to early-phase apoptosis and requires an intact plasma membrane (3). In particular, *in vitro* studies performed by Cohen et al. (3) observed that [^3^H]ML-10 accumulation by anti-Fas antibody-treated Jurkat cells occurred before loss of plasma membrane integrity, as indicated by propidium iodide fluorescence imaging and trypan blue labeling (3). Moreover, subjection of untreated Jurkat T-cells to 3 freeze–thaw cycles, which disrupt plasma membrane integrity, did not result in [^3^H]ML-10 accumulation above normal control (3), suggesting that ML-10 may be able to distinguish cells undergoing apoptosis from necrotic and nonviable cells. This hypothesis is further supported by fluorescence microscopy imaging of HeLa cells (human cervix carcinoma) and CT26 cells (murine colon carcinoma) treated with cisplatin and taxotere, respectively (3). In both cases, increased dansyl-ML-10 accumulation was observed after treatment; however, propidium iodide-positive cells (indicating nonviable cells) did not show fluorescence (3). Thus, in applications where measurement of relative changes in apoptosis at the time of imaging is desired, [^18^F]ML-10 may provide a useful option.

In this paper, we showed 4 example cases of patients with GBM who underwent [^18^F]ML-10 PET scans before and after ∼2–3 weeks of therapy. Findings were interpreted on a case-by-case basis for each patient, with each patient's time-to-radiologic progression serving as the clinical end-point. The imaging schedule for this study paralleled that of Allen et al.'s study (15), in which patients with brain metastases underwent a baseline [^18^F]ML-10 PET scan (before therapy initiation) and a therapy response [^18^F]ML-10 PET scan at ∼2 weeks after therapy initiation. In that study, changes in [^18^F]ML-10 SUV between the BL and the 2-week time-point were found to correlate (r = 0.9) with late changes (∼6–8 weeks after therapy) in the morphology of brain metastases measured using CE-MRI.

For the patients with GBM presented in this study (Figures 1–4), changes between BL and ETA [^18^F]ML-10 PET SUV images, derived by averaging dynamic PET frames acquired over 30 minutes starting at 120 minutes after injection, were not clearly correlated with patient TTP. The differences between studies may be because of several factors. First, the clinical endpoints were different. Second, different tumor types were examined between the two studies (i.e. brain metastases versus GBM). Finally, in the current study, the effects of differences in the blood–brain barrier integrity between imaging time-points has not been investigated. Given the different nature of the tumor types and the difference in therapies, such effects may be different between the two studies.

It should be recognized that the biology of a tumor is dynamic and heterogeneous, with cell death and cell proliferation occurring simultaneously. Thus, although assessment of apoptosis may allow for fast decisions regarding therapeutic efficacy (eg, within a few days of therapy initiation), challenges remain with regard to the ability to reliably predict outcome, particularly in the case of an aggressive cancer such as GBM. For example, one subpopulation of tumor cells may be more sensitive to chemotherapy than others and, therefore, would contribute to a high apoptosis signal, whereas the remaining subpopulations could be refractory to therapy (2). In addition, a time-dependent cell death profile, containing a peak apoptotic response followed by a decline in signal due to a diminishing population of tumor cells available to undergo apoptosis, has been observed in some pre-clinical studies that administered either 1 or 2 doses of chemotherapy and then measured radiolabeled Annexin-V accumulation over time (16–19). These preclinical results suggest that there is likely an optimal time after therapy initiation to image and quantify apoptosis for the purpose of response assessment in patients with GBM.

Several approaches have been suggested to address these potential confounding factors for interpreting therapeutic response using apoptosis imaging, including diffusion-weighted MR imaging (20), perfusion MR imaging (21), and acquiring an ^18^F-labeled fluorodeoxyglucose PET scan (2). In this report, we show another approach to assess the presence of viable tumor cells at the time of [^18^F]ML-10 PET using ^23^Na MRI. A motivation for this approach is based on the observation that TSC levels are generally elevated in malignant brain tumors (22). This increase has been largely associated with reduced Na^+^ efflux by adenosine triphosphate-driven Na^+^/K^+^-ATPase pumps in tumor cells, leading to increased intracellular ^23^Na concentration (22, 23), which has been associated with tumor malignancy and proliferation (10). Several methods have been proposed and are currently under investigation to quantify intracellular ^23^Na concentration (13, 23). In this manuscript, we showed a case (Figure 4) where vBSC was used (13). Changes in this value between scans are currently being investigated as a measure of interval tumor proliferation/regression.

## Conclusions

For the limited number of patients with GBM presented, visual and quantitative interpretation of pre- and post-therapy (∼2–3 weeks) [^18^F]ML-10 SUV images, derived by averaging dynamic PET frames acquired over 30 minutes starting at 120 minutes after injection (the ETA of ML-10 #4 initiated 175 minutes after injection), did not reveal an obvious relationship with TTP. The effects of optimized timing of an early assessment scan and incorporation of additional biomarkers into the analysis have not been fully investigated.
